# Immunopathological characteristics and therapeutic effects of UC-MSCs in a pigeon breeder’s lung mouse model: Immunopathology of PBL and effects of UC-MSCs

**DOI:** 10.3724/abbs.2025010

**Published:** 2025-01-21

**Authors:** Jingran Xu, Li Li, Yaping Zhou, Zulipikaer Abudureheman, Lexin Xue, Chao Wu, Xiaoguang Zou

**Affiliations:** 1 The First Affiliated Hospital of Xinjiang Medical University Urumqi 830000 China; 2 Department of Respiratory and Critical Care Medicine First People′s Hospital of Kashi Kashi 844000 China; 3 Clinical Research Center of Infectious Diseases (Pulmonary Tuberculosis) First People’s Hospital of Kashi Kashi 844000 China; 4 Department of Respiratory and Critical Care Medicine the First Affiliated Hospital of Shihezi University Shihezi 832061 China

**Keywords:** pigeon breeder’s lung, immunopathology, UC-MSC, therapy

## Abstract

Hypersensitivity pneumonitis (HP), including pigeon breeder’s lung (PBL), often progresses from acute inflammation to fibrosis, impairing lung function and limiting targeted therapeutic strategies. Mechanistic studies on PBL progression are limited by the lack of preclinical animal models and a predominant focus on patient data. This study explores the immunopathological characteristics of all stages of PBL in mice and evaluates the therapeutic potential of human umbilical cord-derived mesenchymal stem cells (UC-MSCs) during the non-fibrotic stage. PBL models are created in A/J mice through tracheal instillation of pigeon dropping extract (PDE) protein powder. Different doses (0.4 × 10 ^6^, 0.8 × 10 ^6^, and 1.6 × 10 ^6^ cells per animal) and frequencies (1–2 times) are administered to the model. The immunopathological characteristics of *PBL* and the therapeutic effects of UC-MSCs are assessed using micro-CT, pulmonary function, histopathology, cell counts in BALF, HYP levels, inflammatory factor levels, immunohistochemistry, and fibrosis marker expression in lung tissues. The results show that PDE exposure consistently impairs pulmonary function and increases the levels of inflammation and fibrosis markers as the disease progresses. Model mice experience non-fibrotic stages (acute inflammation) from days 0–36, mild fibrosis from days 37–77, and severe fibrosis from day 78 onwards. UC-MSCs, particularly at the highest dose (1.6 × 10 ^6^ cells), effectively treat non-fibrotic *PBL* by improving pulmonary function (lung ventilation area recovers) and reducing inflammation and fibrosis. This study successfully establishes PBL mouse models reflecting both the acute (inflammatory) and chronic (fibrotic) stages, and UC-MSCs have the potential to delay fibrosis, providing new therapeutic options for PBL and other inflammation-induced lung fibrotic diseases.

## Introduction

Pigeon breeder’s lung (PBL) is the predominant form of hypersensitivity pneumonitis (HP) [1]. It is an immune-mediated interstitial lung disease characterized by bronchial and alveolar inflammation caused by repeated exposure of susceptible individuals to proteins in pigeon droppings [ 2 , 3]. PBL is particularly prevalent in Xinjiang Kashi, which is closely related to the daily hobbies and living habits of local residents. The region not only has a high number of free-range pigeons, leading to frequent human-pigeon contact but also serves as a crucial protein source and a key income for local farmers [4]. Additionally, Kashi has a dry climate, with low annual precipitation and frequent gusty winds and dusty weather [5]. This makes pigeon droppings difficult to condense and settle. As a result, these droppings remain suspended in the air for extended periods, leading to continuous and low-level exposure to bird antigens for both pigeon owners and their neighbors. As a result, the incidence of PBL is increasing, particularly in regions where avian contact is prevalent, such as Kashi, Xinjiang.

PBL can be categorized into two distinct phases: acute/non-fibrotic (inflammation) and chronic/fibrotic (inflammation with fibrosis or fibrosis alone) [6]. The non-fibrotic phase is predominantly inflammatory and often reversible with antigen avoidance, triggered by intermittent high-level antigen exposure [7]. In contrast, the fibrotic phase involves sustained, low-dose antigen exposure, leading to irreversible lung damage characterized by fibrosis [8]. Given the similarities between PBL and other inflammation-induced lung fibrotic diseases, understanding the pathogenesis of PBL is crucial. Without effective intervention, patients in the fibrotic stage face irreversible lung function loss and potential death from respiratory failure if not treated with a lung transplant [9]. To improve the diagnosis and prognosis of PBL patients, it is essential to develop comprehensive disease models that reflect various stages of progression. These models will help identify key pathophysiological features and guide early therapeutic interventions, preventing progression to the irreversible fibrotic stage.

Mesenchymal stem cells (MSCs) have demonstrated potential in the areas of tissue regeneration, anti-inflammation, and anti-fibrosis [ 10, 11]. In particular, human umbilical cord-derived MSCs (UC-MSCs) offer advantage such as wide availability, non-invasive collection, ethical acceptance, and low immunogenicity [12]. Its use in regenerative medicine [13], especially in lung diseases, has increased, including in clinical trials and basic research on idiopathic pulmonary fibrosis (IPF) [ 14, 15]. Given the parallels between PBL progression and lung damage from IPF, UC-MSCs may also have therapeutic potential in PBL.

In the present study, we constructed a PBL mouse model to clarify the immunopathological and fibrotic processes at various stages of disease progression (non-fibrotic/fibrotic). Different doses and frequencies of UC-MSCs were administered during the non-fibrotic stage to evaluate their therapeutic efficacy and determine the optimal dose for early intervention. Furthermore, we investigated the potential of UC-MSCs to delay fibrosis progression, aiming to provide insights for future clinical trials on the use of UC-MSCs as a treatment for PBL and potentially for other lung fibrotic diseases.

## Materials and Methods

### Animals

All animal experiments were approved by the Ethics Committee of Xinjiang Medical University (IACUC-20231010-06) and adhered to ARRIVE (Animal Research: Reporting of *In Vivo* Experiments) guidelines and other relevant protocols. This study was conducted in accordance with relevant guidelines and regulations.

In this study, 8-week-old healthy, male, clean-grade, adult A/J mice were provided by Jiangsu Jicui Pharmachem Biotechnology Company (Nanjing, China) [production license number SCXK (Su) 2023-0009]. The experiments were conducted in the SPF grade animal laboratory of Basic Medical Sciences and Oncology, Chinese Academy of Sciences. All experimental mice were housed under standard conditions with free access to food and water, a temperature of 22 ± 2°C, and a humidity of 60%–80%.

### Construction of PBL mice model

Pigeon dropping extract (PDE) [16] was utilized as an inducer to construct animal models, following the methodology outlined by Li *et al*. [17] and Ohtani *et al*. [18].

A/J mice were randomized to the control group or the PBL group. The methods of constructing the PBL model also referenced Li’s approach [17]. In the PBL group, on days 0–7, 200 μL of PDE (0.04 g/L) was injected intraperitoneally 3 times/week. Beginning on day 8, 200 μL of PDE (0.4 g/L) was administered via intratracheal instillation 3 times/week. In the control group, the mice were kept in the same air environment and received the same volume of saline.

The mice used to study PBL progression were anaesthetized and sacrificed with isoflurane at 7-day intervals from day 9 until day 141. The mice used for the study of the effects of UC-MSCs treatment were observed for 14 days after the final injection of UC-MSCs, and the serum and lung tissue were collected for subsequent testing.

### Evaluation of staging in PBL mice

The diagnostic criteria for pathology and imaging were adopted from the official 2020 ATS HP diagnostic criteria [19]. Additionally, considering the differences in lung volume and tissue density between mice and humans, we used the micro-CT results as a non-invasive auxiliary reference.

The pathological criteria for determining whether PBL was in the non-fibrotic or fibrotic stage were as follows: 1) Non-fibrotic stage pathological features (at least one biopsy site exhibited the following three pathological characteristics): (a) Cellular interstitial chronic inflammation: i) centrilobular (airway-centric); ii) a cellular nonspecific interstitial pneumonia (NSIP)-like pattern; and iii) predominantly lymphocytes. (b) Cellular bronchiolitis: predominantly lymphocytes (lymphocytes > plasma cells), with focal peribronchiolar lymphocyte aggregation that did not exceed the extent of bronchial-associated inflammation, possibly with organizing pneumonia (Masson bodies) and/or foamy macrophages in the terminal air spaces. (c) Poorly formed non-necrotizing granulomas: i) loose epithelioid cells aggregated and/or multinucleated giant cells aggregated, possibly with cytoplasmic inclusions; ii) these granulomas were located in the peribronchial interstitial areas, terminal airspaces, and/or organizing pneumonia (Masson bodies). 2) Pathological features of the fibrotic stage at least one biopsy site must meet either criterion (a) or (b) plus criterion (c): (a) Fibrotic interstitial chronic inflammation: structural distortion, with fibroblastic foci possibly accompanied by subpleural honeycombing and fibrotic NSIP-like patterns. (b) Centrilobular fibrosis might be accompanied by peribronchiolar metaplasia or bridging fibrosis. (c) Poorly formed nonnecrotizing granulomas, possibly with cellular interstitial chronic inflammation, cellular bronchiolitis, and organizing pneumonia.

Imaging criteria: 1) Non-fibrotic stage imaging features (must have at least one feature of lung parenchymal infiltration and one feature of small airway disease, with diffuse distribution): (a) Lung parenchymal infiltration: ground-glass opacities and mosaic attenuation. (b) Small airway disease: ill-defined centrilobular nodules and air trapping. 2) Fibrotic stage imaging features (must have at least one feature of lung fibrosis distribution and one feature of small airway disease): (a) Lung fibrosis usually manifests as irregular linear or reticular opacities with structural distortion, which might include traction bronchiectasis and honeycombing. (b) Small airway disease: ill-defined centrilobular nodules, mosaic attenuation, the “triple-density” sign, and/or air trapping.

### UC-MSCs preparation and treatment dosage

The UC-MSCs used in this study were manufactured by VCANBIO Cell & Gene Engineering Corp. (Tianjin, China) in a GMP facility, which was certified by the National Institutes for Food and Drug Control of China. Briefly, UC-MSCs were obtained according to methods described in a previous study [20]. The UC-MSCs used in our experiments were passage 5 cells. The cells were identified to meet the minimal criteria according to the International Society of Cell Therapy standard [21]. The quality and viability of the UC-MSCs were reconfirmed by a cell counter (C100-SE; REWARD, Shanghai, China) after preparation and before each infusion in PBL mice. UC-MSCs surface markers, including CD19, CD34, CD11b, CD45, CD73, CD105, CD90 and HLA-DR, were identified via flow cytometry (FACS Calibur; BD, Franklin Lakes, USA) and have the potential to differentiate into osteoblasts, adipocytes and chondrocytes ( Supplementary Figure S1).

The treatment dosages for the PBL mice were adapted from a previous study [22] and industry guidance from the US Food and Drug Administration [23]. Therefore, in this study, to assess the therapeutic effects of different amounts of UC-MSCs, the injection doses used were as follows: low-MSCs group (0.4 × 10 ^6^/200 μL/individual), medium-MSCs group (0.8 × 10 ^6^/200 μL/individual), and high-MSCs group (1.6 × 10 ^6^/200 μL/individual). Each dose group was further divided into one-treatment subgroup and two-treatment subgroup. On day 22, one-treatment subgroup was injected with UC-MSCs via the tail vein once. On days 22 and 29, the two-treatment subgroup was injected with UC-MSCs via the tail vein twice consecutively. The same volume of saline was injected into the tail vein of the control group and the model group. The treatment, control and model mice were all observed for 14 days after the last injection. Therefore, the one-treatment subgroup was sacrificed on day 36, and the two-treatment subgroup was sacrificed on day 43.

### Homing of UC-MSCs in the lungs

A DiR staining kit (Macklin, Shanghai, China) was used to assess the lung targeting ability of UC-MSCs according to the manufacturer’s protocol. The mice were injected with DiR-labelled UC-MSCs through the tail vein. Photos were taken at 1, 2, 3, and 12 h after injection via an IVIS Spectrum system (PerkinElmer, Waltham, USA). The major organs (lung, liver, and spleen) were dissected and subjected to *in vitr*o imaging. Living image analysis software was used to quantify the fluorescence intensity of DiR.

### Micro-CT of mice

The mice were placed in an induction box (oxygen flow rate of 0.8–1.5 L/min) and anaesthetized with 1.5%–2% isoflurane. Then, the mice were placed in the supine position (the face was covered with an anesthetic mask for continuous anesthesia) in a micro-CT scanner (NMC-200; PINGSENG Healthcare, Inc., Shanghai, China). Micro-CT was performed at a 60 kV voltage and 130 μA current, and the thoracic lungs of the mice were scanned. The lung structure was reconstructed with Avatar software (version 2.0.11; PINGSENG Healthcare, Inc.), and the 3D slicer software (version 5.6.2) was used for image analysis.

### Pulmonary function assessment

Whole-body plethysmography (WBP; Buxco Research Systems, Wilmington, USA) was used to perform pulmonary function tests. The small animal pulmonary function test used in this study was non-invasive. The mice were placed into the tester, and after the mice were in a stable state with stable respiration, the basal values of respiration were recorded. Changes in respiratory rate (f), enhanced pause (Penh), minute volume (MV), tidal volume (TV), and inspiratory time (Ti), expiratory time (Te), peak expiratory flow (PEF), peak inspiratory flow (PIF), and expiratory flow 50 (EF50) were expressed as averages of 3 min recordings.

### Analysis of cells and inflammatory cytokines in bronchoalveolar lavage fluid (BALF)

BALF was collected as previously described [24]. Then, the BALF was centrifuged (1500 rpm, 10 min), the supernatant was used for cytokine analysis, and the cell sediments were resorted with PBS (1 mL). A hemocytometer was used to count total cells in the BALF, and neutrophil, lymphocyte, eosinophil, and macrophage counts were performed via Wright and Giemsa staining kit (BASO, Zhuhai, China). The levels of TGF-β, TNF-α, MIP-1α, and IL-2 in the BALF were determined using the corresponding ELISA kits (Multi Sciences, Shanghai, China) following the recommended protocols.

### Analysis of C3 and C4 in serum

Peripheral blood (1 mL) was collected via orbital blood sampling and centrifuged for 15 min (3000 rpm, 4°C). Serum was collected and stored at -80°C for measurement. The levels of C3 and C4 in the serum were determined using the corresponding ELISA kits (CUSABIO, Suzhou, China) following the recommended protocols.

### Measurement of the level of lung hydroxyproline

The lung tissue homogenate used was described in a previous study [25]. The hydroxyproline (HYP) content was assessed using an HYP assay kit (Elabsciecne, Wuhan, China) and measuring the absorbance at 560 nm.

### Histology and immunohistochemistry

Paraffin-embedded lung tissue blocks were serially cut into 4-μm-thick sections (3 sections per mouse, 3 mice for each group) according to a previous study [26]. The sections were then stained with hematoxylin and eosin (H&E) and Masson’s trichrome to evaluate the degree of lung inflammation and fibrosis. H&E-stained lung sections were observed under a light microscope (E200; Nikon, Tokyo, Japan) and scored for inflammation on the basis of the degree of infiltration of inflammatory cells around bronchioles, bronchi, blood vessels and pulmonary interstitium [27]. Inflammation was graded as follows: grade 1 (no inflammatory cell infiltration), grade 2 (occasional inflammatory cell infiltration), grade 3 (1–2 layers of inflammatory cell infiltration around bronchioles or vessels), grade 4 (3–4 layers of inflammatory cell infiltration around bronchioles or vessels), and grade 5 (more than 5 layers of inflammatory cell infiltration around most bronchioles or vessels).

The pulmonary fibrosis level was analyzed via the Ashcroft scale (score range, 0–8) [28]. Each lung specimen was blindly scored by three independent pathologists.

For immunohistochemistry (IHC), after antigen repair and blocking endogenous peroxidase, the lung tissue sections were incubated with anti-fibronectin (15613-1-AP; Proteintech, Chicago, USA), anti-collagen I (AF7001; Affinity, Cincinnati, USA), anti-α-SMA (14395-1-AP; Proteintech), anti-MMP-2 (AF5330; Affinity) and anti-TIMP-1 (ab216432; Abcam, Cambridge, UK) antibodies overnight at 4°C, followed by staining with an HRP-conjugated secondary antibody and DAB substrates. Images of the stained sections were captured via light microscopy, and quantitative analysis of the IHC results was performed using ImageJ software (NIH, Bethesda, USA).

### Western blot analysis

Lung tissues were lysed in RIPA buffer containing protease inhibitors. The protein concentration was determined using bicinchoninic acid (BCA; TransGen Biotech, Shanghai, China). Equal amounts of proteins were separated by SDS polyacrylamide gel electrophoresis (SDS-PAGE) and then transferred to PVDF membranes (Millipore, Billerica, USA). After incubation with the appropriate primary and secondary antibodies ( Supplementary Table S1), the protein bands were visualized with a ChemiScope imaging system (Clinx Science Instruments, Shanghai, China).

### Statistical analysis

The statistical analysis and graphing were performed via GraphPad Prism 9 (GraphPad Software, La Jolla, USA). Data are expressed as the mean ± SD. Differences among the groups were analyzed by one-way ANOVA or two-way ANOVA followed by Bonferroni’s multiple comparison test. *P*​< 0.05 was considered statistically significant.

## Results

### CT and pulmonary function characteristics of PBL progression in mice

**Figure 1:**
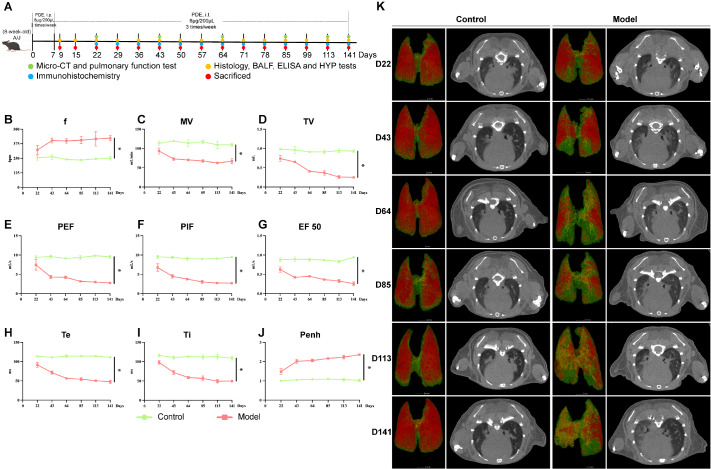
Figure 1 Imaging and pulmonary function characteristics of PBL progression in mice (A) Illustration of the construction of the PBL mouse model. (B–J) Pulmonary function tests were performed on control (n = 3) and model (n = 3) samples on days 22, 43, 64, 85, 113 and 141. Data are expressed as the mean ± SD. *P < 0.05 vs control. (K) Controls (n = 3) and models (n = 3) were subjected to micro-CT to obtain representative lung computed tomography images on days 22, 43, 64, 85, 113 and 141. Image reconstruction of mouse lungs was performed via CT-Avatar software. Red areas represent normal inflation, green areas represent poor inflation, and yellow areas represent no inflation.

To comprehensively and dynamically study the progression of PBL and the characteristics of the non-fibrotic and fibrotic phases, we constructed days 0–141 A/J mouse model using PDE via intratracheal instillation ( Figure 1A). Compared with those of the control mice ( Figure 1B–J), the pulmonary function of the model mice exhibited notable increases in f and Penh over time, whereas TV, MV, PIF, PEF, Ti, Te, and EF50 progressively decreased ( *P* < 0.05). Reconstruction of the lung structure and micro-CT suggested that the texture of both lungs in model mice gradually thickened on days 0–43, with lung inflammation as the main manifestation ( Figure 1K). From day 43 onwards, diffusely distributed patchy ground-glass shadows of varying degrees could be observed in the lung tissues, which gradually worsened. By day 85, lattice-like and honeycomb shadows were evident in the lung tissues. Compared with that in the control group, the normal inflated area of the lungs in the model group gradually decreased.

### Immune cells and inflammatory cytokines are associated with PBL progression in mice

**Figure 2:**
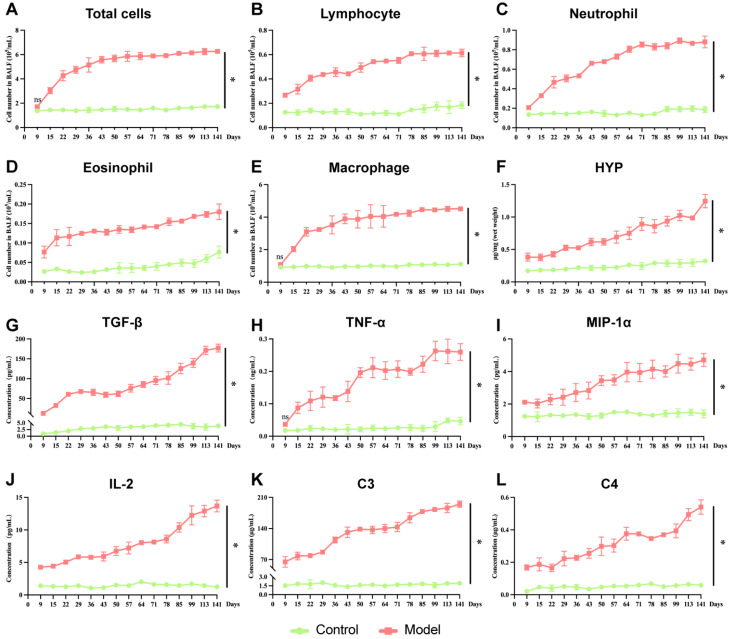
Figure 2 Immune cell and inflammatory cytokine levels during PBL progression in mice (A–F) Cell counts in the BALF and HYP in lung tissues (n = 3). (G–L) ELISA analysis of TGF-β, TNF-α, MIP-1α, and IL-2 in the BALF and C3 and C4 in the serum (n = 3). Data are expressed as the mean ± SD. *P < 0.05 vs control. ns, no significant difference.

Next, to better understand the immune status of the model group throughout disease progression, we assessed the number of immune cells in the BALF, HYP levels in lung tissues, and inflammatory factors in the BALF and serum. Compared with those in the control group, the numbers of total cells, lymphocytes, neutrophils, eosinophils, macrophages and HYP were significantly higher over time ( *P*​< 0.05; Figure 2A–F). The levels of TGF-β, TNF-α, MIP-1α, IL-2, C3, and C4 were also consistently increased ( *P*​< 0.05; Figure 2G–L).

### Histology and immunohistochemistry of PBL progression in mice

**Figure 3:**
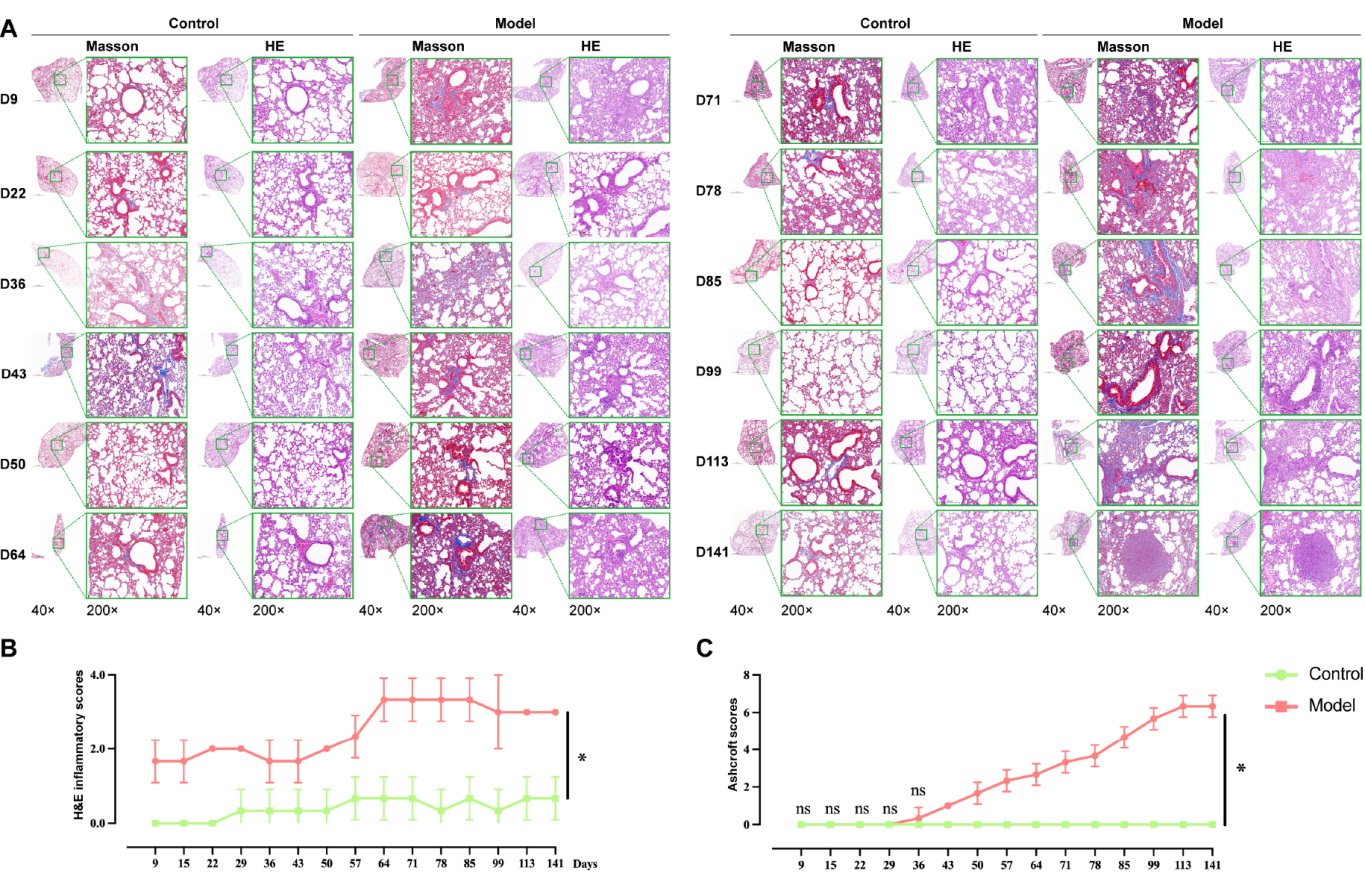
Figure 3 Histology of PBL progression in mice (A) H&E and Masson staining of lung sections (n = 3). Scale bars: 1000 μm and 200 μm, respectively. (B) H&E inflammatory scores. (C) Ashcroft scores. Data are expressed as the mean ± SD. *P < 0.05 vs control. ns, no significant difference.

We next performed histological examinations of the lung tissues. Compared with those in the control group, H&E staining ( Figure 3A) revealed that oedema and inflammatory exudation were the main manifestations in the model group on days 0–42, with significant infiltration of inflammatory cells, disordered lung tissue structure, and rupture and fusion of alveoli. On days 43–70, patchy infiltration of inflammatory cells was observed centered on the bronchioles, and the gradual removal of inflammatory cells was accompanied by new inflammatory exudate. On days 71–141, oedema appeared in the pulmonary interstitium, with inflammatory foci accompanied by numerous epithelioid cells, neutrophil necrosis, and granulomas surrounded by fibrous tissues, with a small amount of fibrous tissue growing into granulomas. H&E staining revealed a significant trend toward higher inflammation scores ( *P *​< 0.05; Figure 3B). Masson staining revealed that the alveolar walls of the models were thickened and locally accompanied by inconspicuous fibrous tissue hyperplasia around the bronchioles on days 0–35 ( Figure 3A). On days 36–77, fibrous tissue deposits were found around the bronchioles and gradually increased in size. Beginning on day 78, the number of fibrous tissue deposits around the bronchioles increased, and fibrous deposition began to appear in the lung interstitium, gradually increasing. The Ashcroft score tended to increase on day 29, with a significant difference between the two groups starting on day 43 ( *P*​< 0.05; Figure 3C).

**Figure 4:**
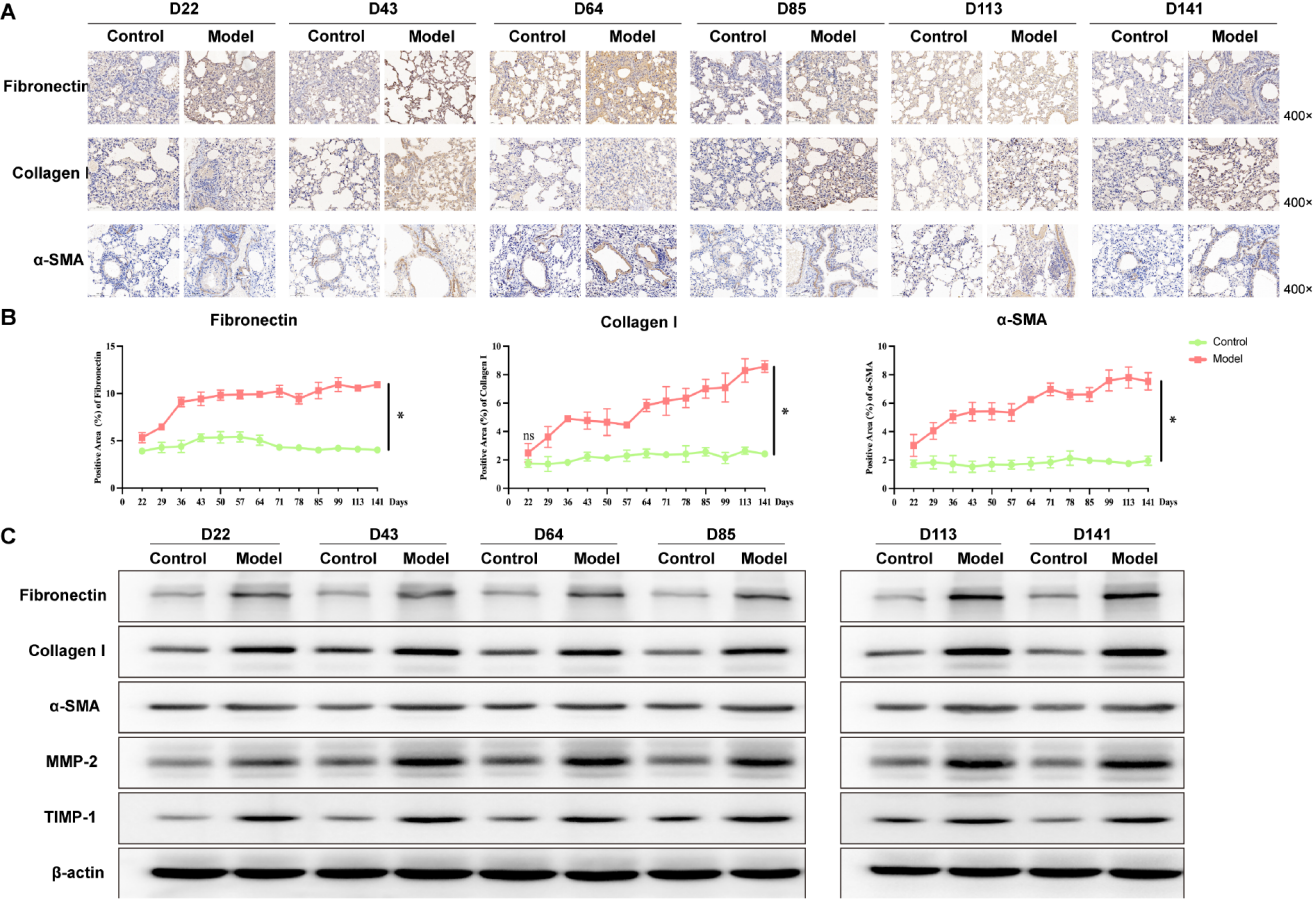
Figure 4 Fibrotic levels of PBL progression in mice (A) IHC staining of lung sections (n = 3). Scale bar: 100 μm. (B) Positive area (%) of fibronectin, collagen I, and α-SMA (n = 3). Data are expressed as the mean ± SD. *P < 0.05 vs control. ns, no significant difference. (C) Western blot analysis of fibronectin, collagen I, α-SMA, MMP-2, and TIMP-1 expressions (n = 3).

On the basis of Masson’s trichrome staining results, we conducted IHC and western blot analysis to evaluate the trends in the expressions of fibrotic markers. The expressions of fibronectin, collagen I, α-SMA, MMP-2, and TIMP-1 were detected by IHC ( Figure 4A and Supplementary Figure S2A), revealing a gradual increase over time ( *P*​< 0.05; Figure 4B and Supplementary Figure S2B). The western blot analysis results ( Figure 4C) were consistent with the IHC results ( *P *​< 0.05; Supplementary Figure S2C).

Consequently, we evaluated disease progression in a PBL mouse model from day 0 to day 141 and concluded that days 0–36 were in the non-fibrotic phase, days 37–77 were in the mild fibrotic phase (fibroplasia confined to the periphery of the bronchi), and from day 78 onwards, they were in the more severe fibrotic phase (fibrotic deposition of interstitial lungs began to occur).

### Therapeutic efficacy of UC-MSCs in PBL mice

**Figure 5:**
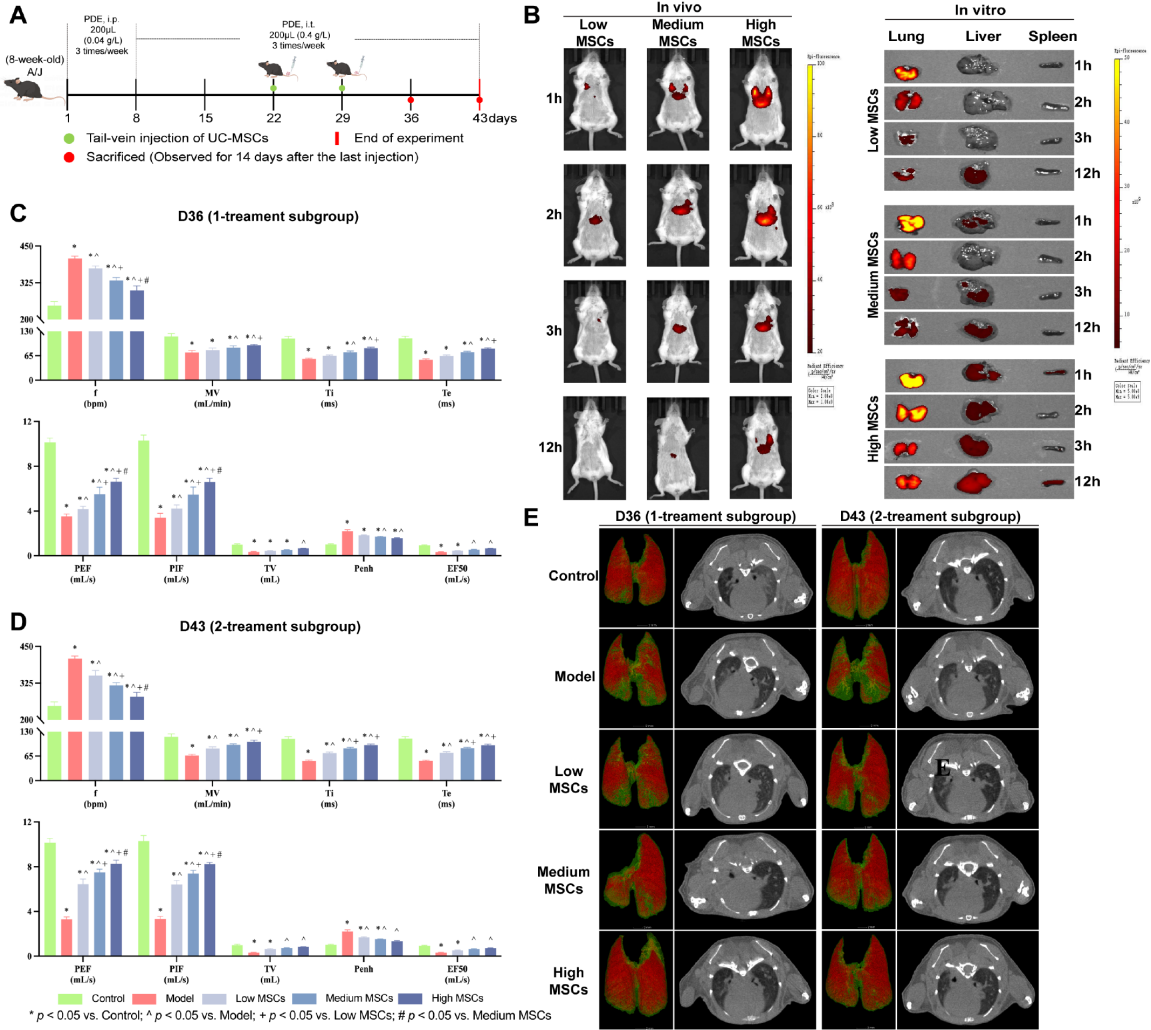
Figure 5 Therapeutic effect of UC-MSCs in PBL mice by micro-CT and assessment of lung function (A) Illustration of the effects of UC-MSCs treatments on PBL model mice. (B) In vivo and in vitro images of UC-MSCs homing in PBL mice after the first injection (n = 3). (C,D) Pulmonary function after UC-MSCs injection (n = 6). Data are expressed as the mean ± SD. * P < 0.05 vs control, ^P < 0.05 vs model, + P < 0.05 vs low MSCs, #P < 0.05 vs medium MSCs. (E) Representative lung computed tomography images obtained via micro-CT after UC-MSCs injection (n = 3). Image reconstruction of mouse lungs was performed via CT-Avatar software. Red areas represent normal inflation, green areas represent poor inflation, and yellow areas represent no inflation.

Following the determination of disease progression stages in PBL mice and inspired by the excellent properties of UC-MSCs, we initiated treatment with UC-MSCs in the non-fibrotic stage of the PBL model. On day 22 after continuous PDE induction to induce the PBL model, low-dose (0.4 × 10 ^6^ cells/200 μL), medium-dose (0.8 × 10 ^6^ cells/200 μL), and high-dose (1.6 × 10 ^6^ cells/200 μL) UC-MSCs were simultaneously administered to one-treatment subgroup and two-treatment subgroup via the tail vein. Afterward, on day 29, the two-treatment subgroup was given the same dose of UC-MSCs as the one-treatment subgroup. The control and model groups were given the same volume of saline each time. All the groups were continuously observed for 14 days after the last treatment, and the mice treated with UC-MSCs once or twice were euthanized on days 36 and 43, respectively ( Figure 5A).

Before the therapeutic effect of UC-MSCs can be evaluated, we need to clarify the lung targeting ability of UC-MSCs *in vivo*, so we chose DiR as a fluorescent probe to label UC-MSCs. Figure 5B showed that the DiR signals *in vivo* exhibited strong fluorescence signals from 1–12 h, and the fluorescence intensity was positively correlated with the number of UC-MSCs injected. Next, we investigated the distribution of fluorescence in the major organs. The intensity of DiR was much greater in the lungs than in the liver and spleen at 1–12 h. Overall, these results indicated that UC-MSCs had a strong ability to home to the lungs of the model group in this study and could remain stable in the lungs for a certain period of time.

Compared with those in the model group, the micro-CT results indicated that the thickening of the texture in both lungs was significantly relieved and that inflammation was attenuated after UC-MSCs treatment ( Figure 5E). With the same treatment frequency, the low, medium, and high doses were all effective. The inflated area almost returned to normal after high-dose treatment. Given that the same dosage of UC-MSCs was used, the outcomes of the one-treatment subgroup and two-treatment subgroup were similar.

The pulmonary function results revealed that UC-MSCs treatment effectively improved lung function ( *P*​< 0.05; Figure 5C,D). With the same number of treatments, the improvement was most significant in the high-dose group ( *P*​< 0.05). In addition, with the same therapeutic dose ( Supplementary Figure S3A–I), the effect of two consecutive treatments was significantly better than that of only one treatment ( *P*​< 0.05).

### The dynamics of histology and inflammatory cytokines in the UC-MSCs treatment group

**Figure 6:**
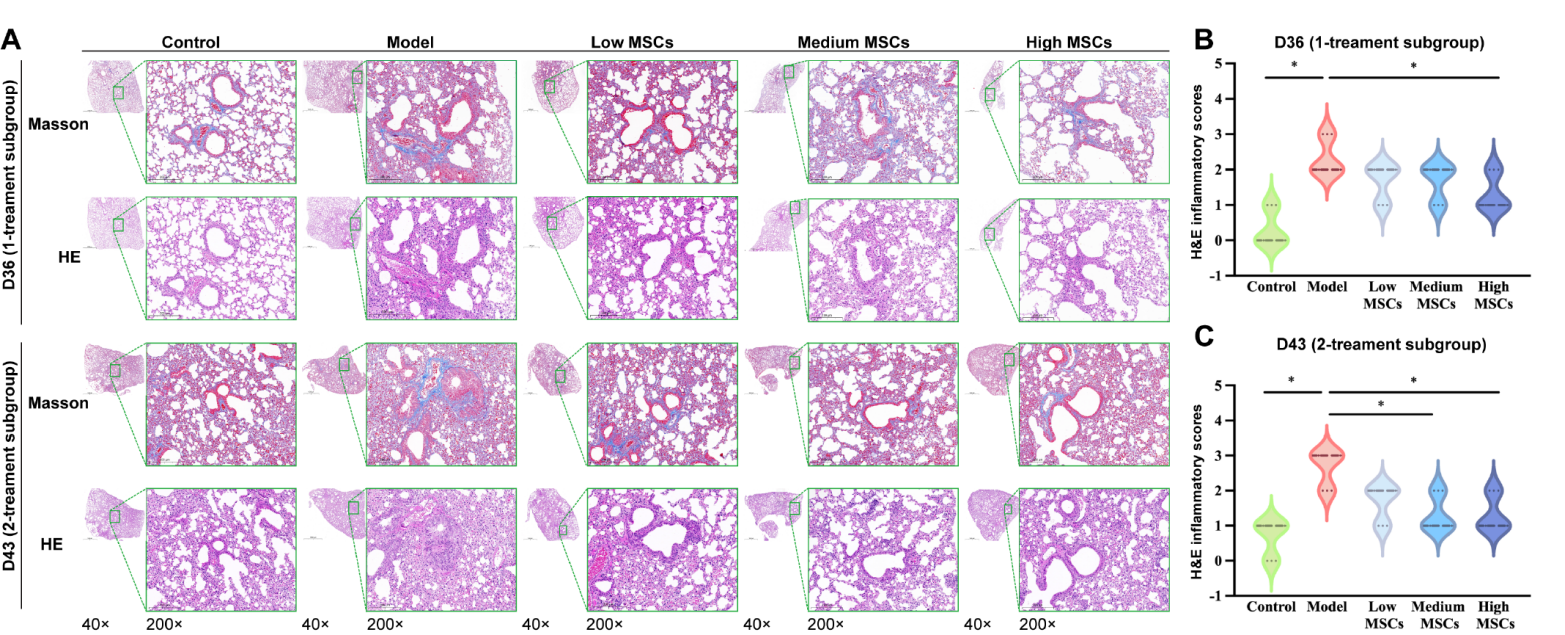
Figure 6 Histology after UC-MSCs treatment (A) H&E and Masson staining (n = 6). Scale bars: 1000 μm and 200 μm, respectively. (B,C) Inflammatory scores (n = 6). *P < 0.05.

We further performed histological examination of the lung tissues. H&E staining and inflammation scores revealed increased inflammatory cell infiltration around the bronchioles in the model group. With the same frequency of treatments ( Figure 6A), histopathological damage to the lungs of the mice was mitigated after treatment with low, medium or high doses of UC-MSCs compared with the model group. Notably, the inflammation scores decreased most significantly in the high UC-MSCs group ( *P*< 0.05; Figure 6B,C). Moreover, at the same therapeutic dose, a notable decrease in the number of inflammatory cells surrounding the bronchioles was observed following two consecutive treatments compared with a single treatment ( Figure 6A). Additionally, inflammation scores were lower in the two-treatment subgroup than in the one-treatment subgroup ( Supplementary Figure S4C), with no difference between the groups ( *P*​> 0.05). Masson staining revealed that fibrous tissue proliferation in the lung tissue was not significant in any of the groups ( Figure 6A). It was slightly more pronounced on day 43 than on the early days. Ashcroft scores were only slightly higher in the model group than in the control group on day 43 ( Supplementary Figure S4A,B), but were not significantly different ( *P*> 0.05; Supplementary Figure S4D).

**Figure 7:**
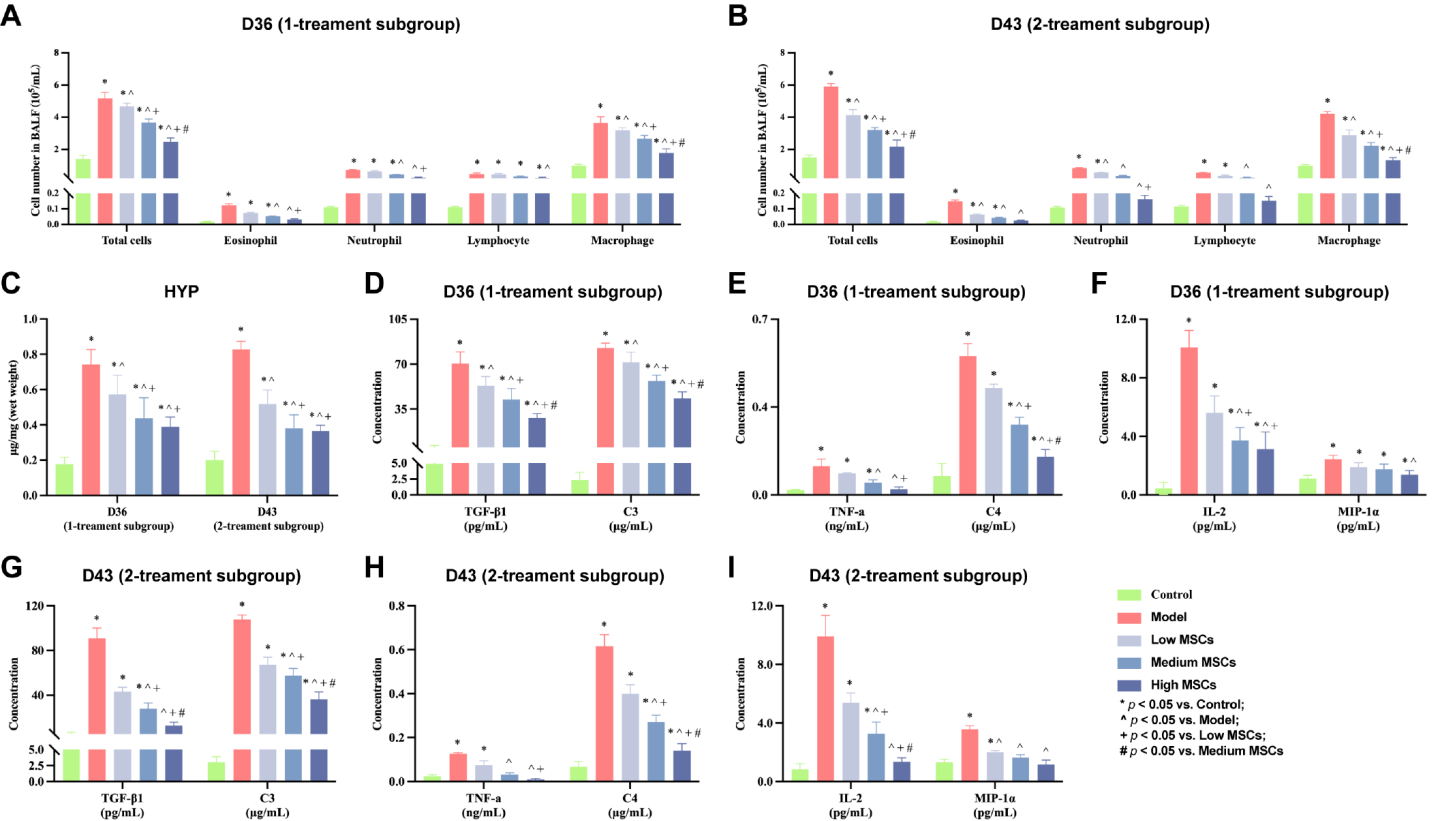
Figure 7 Inflammatory cytokines after UC-MSCs treatment (A,B) Cell counts in the BALF (n = 6). (C) HYP levels in the lung tissues (n = 6). (D–I) ELISA analysis of TGF-β, TNF-α, MIP-1α, and IL-2 in the BALF and C3 and C4 in the serum (n = 6). Data are expressed as the mean ± SD. *P < 0.05 vs control, ^P < 0.05 vs model, +P < 0.05 vs low MSCs, # P < 0.05 vs medium MSCs.

UC-MSCs significantly reduced immune cell, HYP and inflammatory cytokine levels in the BALF and serum. In the one-treatment subgroup, the low, medium, and high UC-MSCs treatments effectively reduced the inflammatory response compared with the model group ( *P*​< 0.05; Figure 7A,D–F). There was no difference in neutrophil, eosinophil, or TNF-α levels following high UC-MSCs treatment compared with those in the control group ( *P*​> 0.05). Similarly, in the two-treatment subgroup, all doses of UC-MSCs effectively reduced inflammation compared with the model group ( *P*​< 0.05; Figure 7B,G–I). Compared with those in the control group, the neutrophils, lymphocytes, TNF-α, and MIP-1α levels in the medium-dose group did not differ ( *P* > 0.05). In the high-dose group, the levels of neutrophils, lymphocytes, eosinophils, TGF-β, TNF-α, IL-2, and MIP-1α did not significantly differ from those in the control group ( *P*> 0.05).

On the other hand, the number of immune cells in the BALF after two consecutive treatments was significantly lower than that after only one treatment ( Supplementary Figure S4F), with the same treatment frequency ( *P*< 0.05). Compared with the one-treatment subgroup, the two-treatment subgroup presented lower levels of HYP and inflammatory factors ( Figure 7C and Supplementary Figure S4E,G), but the difference between the groups was not statistically significant ( *P* > 0.05).

### Effects on fibrosis progression after UC-MSCs treatment

**Figure 8:**
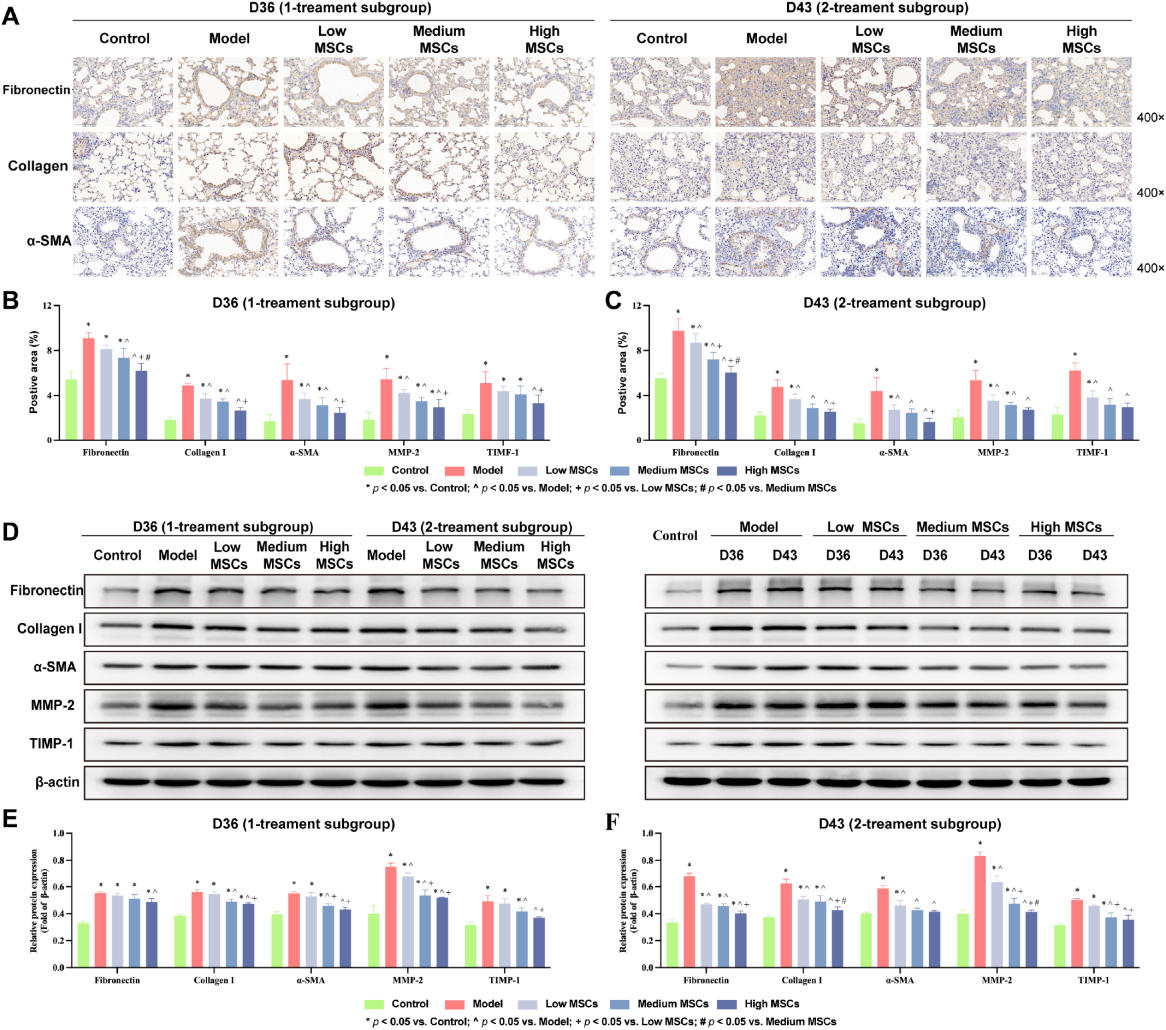
Figure 8 Effects on fibrosis progression after UC-MSCs treatment (A) IHC staining of lung sections (n = 6). Scale bar: 100 μm. (B,C) Positive area (%) of fibronectin, collagen I, α-SMA, MMP-2, and TIMP-1 (n = 6). (D–F) Western blot analysis of fibronectin, collagen I, α-SMA, MMP-2, and TIMP-1 expressions (n = 3). Data are expressed as the mean ± SD. * P < 0.05 vs control, ^P < 0.05 vs model, + P < 0.05 vs low MSCs, #P < 0.05 vs medium MSCs.

Next, we evaluated the therapeutic effects of UC-MSCs by IHC and western blot analysis. IHC showed that UC-MSCs treatment significantly reduced the expressions of fibronectin, collagen I, α-SMA, MMP-2, and TIMP-1 ( *P* < 0.05; Figure 8A and Supplementary Figure S5A), with the most pronounced effect in the high UC-MSCs group. In the one-treatment subgroup, low, medium, and high doses were effective compared with the models ( *P* < 0.05; Figure 8B). No significant differences in the levels of fibronectin, collagen I, α-SMA, or TIMP-1 were detected between the high-dose group and the control group ( *P*​>0.05). In the two-treatment subgroup, the low, medium, and high treatments were also effective compared with the model group ( *P* < 0.05; Figure 8C). Compared with those in the controls, there was no difference in the collagen I, α-SMA, or TIMP-1 levels after treatment in the medium-dose group ( *P* > 0.05), and no difference in the expression of the five proteins after high UC-MSCs treatment was detected ( *P* > 0.05). With the same therapeutic dose, the area of five proteins positive after two consecutive treatments was significantly lower than that after only one treatment, but the difference between the groups was not statistically significant ( Supplementary Figure S5B).

Western blot analysis revealed that the expressions of fibronectin, collagen I, α-SMA, MMP-2, and TIMP-1 were consistent with the IHC results after UC-MSCs treatment ( Figure 8D). Under the same treatment frequency, compared with those in the model group, all 5 proteins in the lung tissue tended to decrease after treatment with 3 doses of UC-MSCs, and the decrease was most obvious in the high-dose group ( *P* < 0.05; Figure 8E,F). Providing the same therapeutic dose, the two-treatment subgroup had a more obvious effect than the one-treatment subgroup ( Supplementary Figure S5C).

## Discussion

In the present study, we successfully constructed a mouse model that encompasses the entire disease cycle (non-fibrotic/fibrotic phase) of PBL, dynamically observing and describing the immunopathological characteristics of lung tissues. We also evaluated the effects of repeated exposure to PDE on the onset and progression of PBL. The results demonstrated that inflammation, pathological histology, and fibrosis markers remained consistently elevated throughout disease progression. Specifically, days 0–36 corresponded to the non-fibrotic phase, days 37–77 corresponded to the mild fibrotic phase (fibrosis confined to peri-bronchiolar areas), and more severe fibrotic changes appeared from day 78 onwards. Notably, UC-MSCs treatment in the non-fibrotic stage resulted in optimal results at a high dose of 1.6 × 10 ^6^ cells per individual, with the subgroup receiving two consecutive treatments performing better than the one-treatment subgroup.

The increasing incidence of PBL and the lack of a comprehensive understanding of its pathogenesis underscore the importance of the use of animal models to study disease progression. A review of the literature from 2001 to 2024 on PBL and bird fancier lungs revealed a focus on epidemiology [29], clinical features (clinical manifestations, immunity, pathology) [ 30, 31], diagnostic markers [ 32– 36], treatments [ 37, 38], and prognosis [ 39, 40]. However, few studies have used animal models to explore disease mechanisms across different stages, especially in relation to chronic exposure to airborne particles from pigeon droppings. The lack of models representing both the non-fibrotic and fibrotic stages has hindered dynamic observations of lung pathology and investigations into the precise mechanisms of disease progression. In this study, we successfully developed a PDE-induced PBL mouse model spanning 0–141 days, representing both the acute (non-fibrotic) and chronic (fibrotic) stages. We dynamically observed changes in lung function, histopathology, inflammation, and fibrosis markers at seven-day intervals. The progression of the model closely resembled the clinical presentation of PBL in humans, with similar pulmonary pathology and elevated inflammatory markers. This model provides valuable insights into the full pathogenesis of PBL and serves as a robust platform for studying the impact of continuous exposure to both antigens on lung diseases.

Currently, antigen avoidance is considered a key intervention for both acute (non-fibrotic) and chronic (fibrotic) PBL [41]. However, the challenge of defining effective avoidance strategies remains [42]. Although studies, such as those by Okuda *et al*. [43], have shown significant prognostic benefits from antigen avoidance in hospitalized patients, others have reported that while it improves lung function in non-fibrotic patients, it does not significantly reduce mortality in either the non-fibrotic or fibrotic stages [44]. Additionally, even after bird removal and environmental cleanup, bird antigens can persist in dust samples and patients’ bodies for extended periods [ 45, 46]. In a study of Nishida *et al*. [44], two of four patients in the non-fibrous phase PBL experienced relapse and continued to live in the same house after the birds were removed. According to previous reports [ 19, 47], PBL is caused not only by direct contact (breeding) with pigeons (and other birds) but also by sustained low-intensity exposure to bird antigens, such as exposure to feather products, wild birds, and neighboring domesticated birds [48]. This highlights the challenge of eliminating chronic low-level antigen exposure, particularly in regions such as Kashi, Xinjiang, where pigeon breeding is both a hobby and a livelihood. In such cases, complete antigen avoidance is nearly impossible, necessitating the development of alternative therapeutic strategies to halt or slow the progression from the non-fibrotic to the fibrotic stage.

The transition from acute (non-fibrotic) to chronic (fibrotic) PBL is resulted from ongoing antigen exposure, which leads to the accumulation of inflammatory cells and cytokines [ 49 , 50], triggering fibroblast activation and fibrosis [51]. UC-MSCs have been demonstrated to potentially suppress inflammatory responses by modulating macrophage and lymphocyte activity [52] through the release of various cytokines and chemokines [ 53, 54]. This results in reduced inflammation and fibrosis, as UC-MSCs can inhibit macrophage polarization [55], regulate metalloproteinase activity, and reduce collagen deposition [56]. Numerous studies have shown that UC-MSCs are particularly effective at reducing lung inflammation and preventing fibrosis when administered during the acute phase of lung injury [ 26 , 57, 58]. In our study, the administration of UC-MSCs in the non-fibrotic (acute) phase not only improved lung function but also reduced inflammatory cell infiltration, decreased the number of inflammatory cells, and lowered the levels of inflammatory factors. Additionally, the levels of collagen deposition and fibrosis markers (HYP, fibronectin, collagen I, α-SMA, MMP-2, and TIMP-1) were reduced. The medium- and high-dose groups presented significantly better outcomes than did the low-dose group, with the best results observed in the high-dose group after two consecutive treatments. These findings suggest that high-dose UC-MSCs treatment may delay fibrosis progression.

Nevertheless, this study has several limitations. Owing to the lengthy modeling time required for fibrotic models, this study focused only on the treatment of non-fibrotic models. Further research is needed to evaluate the therapeutic effects of UC-MSCs in fibrotic models. Additionally, the mechanisms underlying UC-MSCs treatment were not explored in this study and will be investigated in future research.

In summary, we successfully established a PDE-induced PBL mouse model representing both non-fibrotic and fibrotic stages. The model allowed us to evaluate the immunopathological characteristics at different disease stages. UC-MSCs demonstrated therapeutic potential in treating non-fibrotic PBL, improving lung function, reducing inflammatory infiltration, and slowing fibrosis progression. Our findings offer promising insights for future clinical applications of UC-MSCs, particularly in regions where chronic antigen exposure contributes to lung disease progression.

## Supplementary Data

Supplementary data is available at *Acta Biochimica et Biophysica Sinica* online.
